# Synthesis and Evaluation of PEG-PR for Water Flux Correction in an In Situ Rat Perfusion Model

**DOI:** 10.3390/molecules25215123

**Published:** 2020-11-04

**Authors:** Guo Chen, Xingqi Min, Qunqun Zhang, Zhiqiang Zhang, Meiqiang Wen, Jun Yang, Meijuan Zou, Wei Sun, Gang Cheng

**Affiliations:** 1 Department of Pharmaceutics, School of Pharmacy, Shenyang Pharmaceutical University, Shenyang 110016, China; chenguo90s@hotmail.com (G.C.); xingqi_218522@hotmail.com (X.M.); zqq1533346@hotmail.com (Q.Z.); zzq0325@hotmail.com (Z.Z.); MeiqiangWen@hotmail.com (M.W.); yangjun_1995@hotmail.com (J.Y.); zoumeijuansyphu@outlook.com (M.Z.); 2 Department of Biomedical Engineering, School of Medical Devices, Shenyang Pharmaceutical University, Shenyang 110016, China; sunwei@syphu.edu.cn

**Keywords:** phenol red, water flux correction, PEGylation, permeability, everted gut sac (EGS), in situ perfusion model

## Abstract

Phenol red (PR) is a widely used marker for water flux correction in studies of in situ perfusion, in which intestinal absorption usually leads to the underestimation of results. In this paper, we propose a novel marker polyethylene glycol (PEG)-PR (i.e., PR modified by PEGylation) with less permeability and evaluate its application in an in situ perfusion model in rats. PEG-PR was synthesized by the chemical conjunction of polyethylene glycol-4k/5k (PEG-4k/5k) and PR. The synthesized PEG-PR was then characterized using ^1^H-NMR, ^13^C-NMR, ultraviolet (UV), X-ray diffraction (XRD), and differential scanning calorimetry (DSC) analyses. The low permeability of PEG-PR was assessed using everted gut sac (EGS) methods. The apparent permeability coefficients (*P*_app_, 3–8 × 10^−7^ cm/s) of PEG4k/5k-PR exhibited a nearly 15-fold reduction compared to that of PR. The different concentrations of PEG4k/5k-PR did not contribute to the *P*_app_ value or cumulative permeable percentage (about 0.02–0.06%). Furthermore, the larger molecular weight due to PEGylation (PEG5k-PR) enhanced the nonabsorbable effect. To evaluate the potential application of the novel marker, atenolol, ketoprofen, and metoprolol, which represent various biopharmaceutics classification system (BCS) classes, were selected as model drugs for the recirculation perfusion method. The water flux corrected by PEG4k/5k-PR reflected the accuracy due to the nonabsorbable effect, while the effective intestinal membrane permeability (*P*_eff_) of atenolol corrected by PEG4k/5k-PR showed a statistically significant increase (*p* < 0.05) in different intestinal segments. In conclusion, PEG-PR is a promising marker for the permeability estimation when using the in situ perfusion model in rats.

## 1. Introduction

Oral administration remains a priority for drug delivery. Although multiple factors contribute to the complexity of this process, recent developments in biopharmaceutics classification system (BCS) theory have recognized two key parameters—the solubility and intestinal permeability of a drug—that govern the fraction of dose absorbed (F_abs_) [1]. As they have been widely accepted and applied in academic research and industrial regulations, the important status of these two parameters is evident [2]. However, achieving effective intestinal membrane permeability (*P*_eff_), which can be employed to quantify drug permeability in humans, inevitably leads to complicated issues, such as involving ethics and extremely challenging experimental conditions [3]. The lack of data in human trials has driven the development of various models to predict the human intestinal absorption of drugs [4,5,6,7]. Among these models, the in situ perfusion model in rats has been considered as an optimal alternative for drug permeability estimation, as it can preserve the real and bioactive intestinal environment to the utmost extent—something which other in vitro modeling techniques (e.g., based on Caco-2, MDCK, and use of chamber, among others) fail to achieve—and provide good estimates of human intestinal permeability [8,9,10,11,12].

The in situ perfusion model estimates the *P*_eff_ value by measuring the remaining drug in the intestinal perfusion solution in an open loop enteric cavity (SPIP), closed loop enteric cavity (formed by a syringe with the Doluisio method), or peristaltic pump (recirculation method) connected with both ends of an intestinal segment via cannulas and ligation at sampling time points [13,14,15]. As these methods have been continuously modified and verified, extensive drug *P*_eff_ values in different intestinal segments, and even in the colon, have been reported, further providing numerous and reliable sources of information for studying the segmentation- and/or pH-dependent permeability of some compounds [16,17,18,19]. The recirculation method, which allows for a longer retention time of the drug perfusion solution for absorption, is more appreciable for poorly absorbed drugs. Thus, it has been recommended that drugs with low permeability (as determined by SPIP) should be re-evaluated using the recirculation method [20].

Nevertheless, in the calculation of *P*_eff_ using the in situ perfusion model, the volume reduction of the perfusion solution (up to 20%) due to water absorption inevitably introduces error into the measurement of the remaining drug concentration in the intestine, thus leading to inaccurate results [21]. Ultimately, this could invalidate the BCS classification and clinically relevant concentrations determined for some drugs that have a narrow therapeutic window (NTW). Although gravimetric methods and nonabsorbed markers, such as phenol red (PR) and ^14^C-PEG (polyethylene glycol), have been used for correcting water flux through the intestinal segments and a comprehensive validation of these methods has been conducted, some concomitant problems remain unresolved [22,23,24]. For the nonabsorbable markers, ^14^C-PEG has underlying security problems and is not available in general laboratory conditions, while PR may interfere with the transport of some compounds and lead to the underestimation of *P*_eff_ values due to its own non-negligible absorption [22,25,26]. To solve the aforementioned issues, a novel marker, which was synthesized by the conjunction of PR and PEG-4k (classified by the FDA as a zero-permeability substance) [27], was proposed and preliminarily evaluated in SPIP. Previous study results regarding this marker have revealed its promising development prospects [28]; however, the specific performance and application of PEG-PR in an in situ perfusion model have not yet been studied in detail.

**Figure 1:**
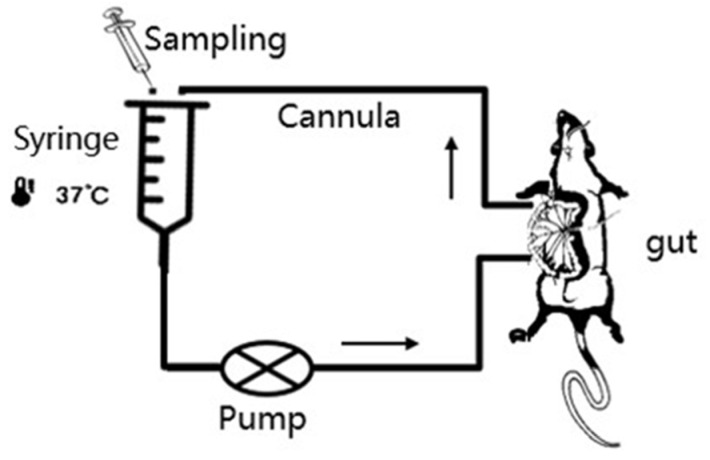
Recirculation method in a rat model.

Therefore, in this work, we synthesized and characterized the PEG4k/5k-PR novel markers, using PEG of different molecular weights. The intestinal permeabilities of different nonabsorbed markers were compared using the everted gut sac (EGS) method, and the permeability mechanisms of PR and PEG4K/5K-PR were elucidated. The application potential of the novel marker was then evaluated by surveying the water flux and the *P*_eff_ corrected by different markers of three model drugs (atenolol, ketoprofen, and metoprolol, classified according to various BCS classes) in intestinal segments and the whole small intestine using the recirculation technique, as shown in Figure 1.

## 2. Materials and Methods

### 2.1. Materials

Phenol red (98%, pure), cresol red (98%, pure), p-toluenesulfonyl chloride (99%, pure) and mPEG (polyethylene glycol monomethyl ether, average *Mn*: 4000 and 5000) were purchased from Sigma-Aldrich Co., LLC (Beijing, China). Atenolol (99%, pure), metoprolol (99%, pure), and phenytoin sodium (99%, pure) were provided by Wuhan Dahua Pharmaceutical Co., LTD (Wuhan, Hubei, China). Ketoprofen (99%, pure) was provided by Hubei Xunda Pharmaceutical Co., LTD (Wuxue, Hubei, China). Ranitidine (99%, pure) was provided by Jiangsu Zhengji Pharmaceutical Co., LTD (Huaian, Jiangsu, China). Ibuprofen (99%, pure) was purchased from Liaoning Pharmaceutical Materials Co., LTD (Shenyang, Liaoning, China). Both solvents of acetonitrile and methanol (Merck KGaA, Darmstadt, Germany) for high performance liquid chromatography (HPLC) analysis were of HPLC grade. All other chemicals were of analytical reagent grade.

### 2.2. Animals

Male Wistar rats (about 200 g) were provided by the Experimental Animal Center of Shenyang Pharmaceutical University. All animal experiments were approved by the Ethics Committee of Animal Experimentation of Shenyang Pharmaceutical University, Shenyang, China. (SYPU-IACUC-C2017).

### 2.3. Synthesis of PEG-PR

**Scheme 1:**
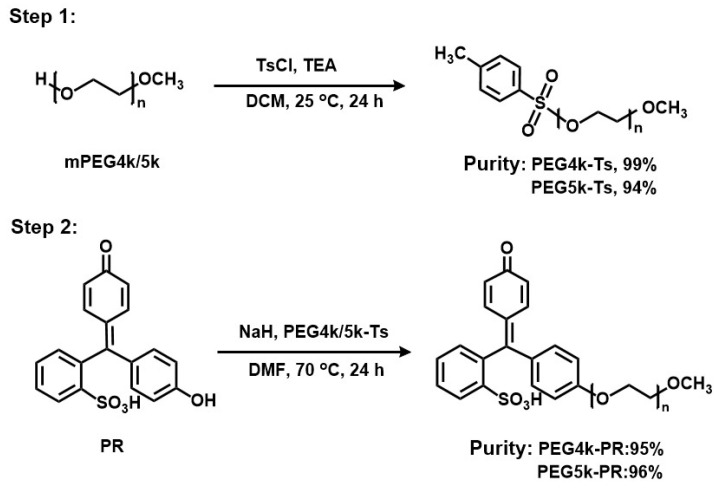
Synthetic route of polyethylene glycol (PEG)-PR.

Synthesis of the PEG-PR was carried out through nucleophilic reaction in two steps, as shown in Scheme 1. To synthesize the intermediate product, 5-mmol mPEG4k/5k, 3-g (16-mmol) p-toluenesulfonyl chloride (TsCl), and 80-mL dichloromethane (DCM) were added sequentially in an eggplant-type flask containing a magnetic stirrer. The eggplant-type flask was then sealed with nitrogen, and 22 mL (159 mmol) of triethylamine (TEA) was added into the container by syringe with stirring at 25 °C.

After 24 h of reaction, the pH value of the reaction mixture was adjusted to 4.0 with HCl (1 mol/L). The reaction mixture was extracted with brine and DCM. The organic layer was collected and concentrated after washing with brine and drying by magnesium sulfate. The product PEG4k/5k-TsCl was finally obtained by precipitation in cold diethyl ether, filtration, and then vacuum drying.

Then, 0.06-g (1.5-mmol) NaH and 0.452-g (1.2-mmol) PR were dissolved in dimethylformamide (DMF). The solution was stirred in an ice bath for 20 min, and PEG4k/5k-Ts was added to the solution dropwise with the protection of nitrogen. The solution was transferred to a 70 °C oil bath for reaction (24 h). Similarly, the reaction mixture was extracted and washed with brine. Then, a yellow powder was obtained by drying, concentration, precipitation, filtration, and vacuum drying. The crude product was finally purified by silica column chromatography eluted with DCM/methanol (90:10–20:80, *v*/*v*).

### 2.4. Characterization of PEG-PR

Structures of the intermediate and final products were confirmed by nuclear magnetic resonance (^1^H-NMR and ^13^C-NMR) spectra (BRUKER 600 UltraShield^TM^, Karlsruhe, Germany). The UV absorption spectrum was investigated using a UV spectrometer (UV-1800) with scanning from 200 to 600 nm. The X-ray diffraction (XRD) pattern was obtained using an X-ray diffractometer (DX2700) with Cu target at 40 kV 30 mA with scanning from 5 to 40° (diffraction angle 2θ) and a step size of 0.03°. Differential scanning calorimetry (DSC) experiments of PR, PEG4k/5k, and PEG4k/5k-PR were conducted using a DSC (DSC1, MettlerToledo, Zurich, Switzerland). Samples were heated from 30 to 350 °C at a rate of 10 °C/min in a nitrogen atmosphere at a flow rate of 80 mL/min.

### 2.5. Stability of PR and PEG4K/5k-PR

To assess the stability of PR and PEG4k/5k-PR, as well as the interplay between the markers and model drugs, Krebs–Ringer buffer (20 mL) was used as a perfusion solution containing PEG4k/5k-PR (56 μM) and model drug (100 μM), which were added into a standard glass reaction vessel with a shaker bath at 37 °C. At the beginning and end (120 min) of the experiment, perfusion solution samples (0.4 mL) were acquired and centrifuged at 12,000 rpm for 10 min to determine the concentration changes of all compounds by HPLC analysis.

### 2.6. Permeability of PEG4K/5k-PR in EGS

The in vitro permeabilities of PR and PEG4k/5k-PR were evaluated by EGS based on a previously described method [29,30]. Briefly, the male Wistar rats were fasted for 1 day with access to only double-distilled water and then anesthetized with ether. Then, the duodenum, jejunum, ileum, and colon (10–15 cm) were cut out and preserved in cold Krebs–Ringer buffer. After the intestinal residue was removed, the intestinal segments were everted by a glass rod and ligated onto the appropriate glass tube. The everted intestinal segments were then injected with Krebs–Ringer buffer prewarmed to 37 °C as a blank and incubated in a donor chamber containing a PR or PEG4k/5k-PR solution of a certain concentration pumped with O_2_ and CO_2_ gases in a 37 °C water bath. Then, 0.4 mL of receiver fluid was collected from the everted intestinal segments, and an equal blank Krebs–Ringer buffer was replenished every 15 min during the experiment.

An internal standard (cresol red 100 μg/mL) was added to every 300-μL intestinal fluid sample. The mixture was vortexed for 30 s and dried under nitrogen blowing at 40 °C. The residue was redissolved in 150-μL methanol and vortexed for 30 s. The supernatant was then subjected to detection by HPLC after centrifuging at 10,000 rpm for 10 min.

The cumulative permeable amount of compound (Q) during the predetermined time, cumulative absorption percentage (W), and apparent permeability coefficient (*P*_app_) were calculated according to the following equations:

(1) $$Q=C_{n}V+0.4\overset{n-1}{\underset{i=1}{\sum }}C_{i}$$

(2) $$P_{app}=\frac{dQ/dt}{AC_{0}}$$

(3) $$w=\frac{Q}{VC_{0}}$$

where *V* represents the volume of blank Krebs–Ringer buffer in the gut, *C_n_* represents the remaining compound concentration in the gut at a given time point, *C*_0_ represents the initial compound concentration in the donor chamber, *dQ/dt* represents the permeable rate, and *A* represents the effective surface area of the gut.

### 2.7. Evaluation of PEG4k/5k-PR with the In Situ Perfusion Model

#### 2.7.1. The Recirculation Perfusion Method

The effective intestinal membrane permeability (*P*_eff_) of each model drug was surveyed using the recirculation intestinal perfusion method, as previously mentioned. A male rat weighing about 200 g was anesthetized and then fixed on a plate heated by a lamp at 37 °C. Through careful operation, two glass tubs were tightly secured on both ends of the intestinal segment using silk sutures. The Krebs–Ringer buffer was injected into the gut from the head end of the intestinal segment with a syringe until the outflow was clear. The intestine was then covered with cotton gauze wetted with 37 °C Krebs–Ringer buffer to maintain intestinal integrity and activity. The peristaltic pump and donor chamber (syringe) were connected with two catheters ligated onto the two abovementioned glass tubs. The intestinal segments were placed carefully back into the rat abdomen to avoid interference with blood circulation. After completion of surgery, 20 mL (V’) of Krebs–Ringer buffer perfusion solution containing the nonabsorbed markers (PR/PEG4k-PR/PEG5k-PR, C’) was added into the donor chamber with a 37 °C water bath to correct the water flux. After stabilizing the whole system for 10 min with the perfusion solution (peristaltic pump, flow: 5 mL/min), the initial sample (0.4 mL, C’_0_) was collected from the donor chamber, and the equal perfusion solution with the model drug (100 μM, C_0_) was added into the donor chamber. Samples (0.4 mL, C’_t_ and C_e_) were then collected every 10 min for 60 min. Finally, the length of the perfused intestinal segment was precisely measured at the end of the experiment.

Internal standard solution (cresol red, 600 μg/mL) was added into the 300-μL sample solution and vortexed for 30 s. Then, 600 μL of methane was added, and the samples were vortexed for 2 min. After homogeneous mixing, the mixture was centrifuged at 10,000 rpm for 10 min. Finally, the 20-μL treated supernatant was subjected to detection by HPLC.

As previously mentioned, the water flux through the membrane due to water absorption and secretion was of such significance that it could have influence on the accuracy of the experimental results. Thus, it was necessary to correct the volume of the perfusion solution during the experiment [21]. In this work, the volumes of the perfused solutions (*V_t_*) were calculated from the experimental concentrations of the markers (*C’_t_*) as

(4) $$V_{0}=\frac{C^{\prime }V^{\prime }}{C_{0}^{\prime }}$$

(5) $$V_{t}=\frac{\left(V_{0}-0.4\right)C_{t-1}^{\prime }+0.4C^{\prime }}{C_{t}^{\prime }}\;\left(t=1,2,3\ldots \right).$$

The corrected drug concentration (*C_t_*) was calculated as

(6) $$C_{t}=\frac{C_{e}V_{t}}{V_{0}}$$

where *C_t_* represents the concentration of the compound corrected by the marker at time *t*, and *C_e_* represents the experimental drug concentration value.

The apparent absorption rate coefficients (*k_a_*) were obtained by the equation of luminal corrected concentration versus time:

(7) $$C_{t}=C_{0}\times e^{-k_{a}\cdot t}.$$

The *P*_eff_ value was obtained from *k_a_* using the following equation, where *R* is the effective radius of the intestinal segment:

(8) $$P_{eff}=K_{a}\cdot R/2.$$

#### 2.7.2. Validation of PR and PEG4k/5k-PR for Water Flux (J_water_) Correction

*J*_water_ corrected by the marker was calculated using the following equation:

(9) $$J_{water}=\frac{d\left(V_{0}-V_{t}\right)}{dt}\;\left(t=1,2,3\ldots \right).$$

### 2.8. Analytical Methods

The sample concentrations were analyzed using an HPLC system (Shimadzu, Kyoto, Japan) consisting of a LC-10Avp liquid chromatograph, SPD-10Avp UV detection, and a Diamonsil C_18_ column (4.6 × 250 mm, 5 μm, Dikma) with a column oven maintained at 30 °C and with a 1.0 mL/min flow. The HPLC conditions of each compound (with isocratic elution) are displayed in Table 1. The precision and accuracy of the analytical methods were validated.

### 2.9. Statistical Analysis

All experimental data of the EGS and the in situ perfusion model are shown as mean ± standard deviation (SD) of parallel trials, with *n* representing the number of used animals. The Statistic Package for the Social Science (SPSS) software version 17 was employed to analyze the data. The assumption of normality from the prerequisites of the parametric tests was assessed using the Shapiro–Wilk test, and the differences between two groups were analyzed using the *t*-test. *p* < 0.05 was considered to indicate statistical significance.

## 3. Results and Discussion

### 3.1. Synthesis and Characterization of PEG-PR

The PEG-Ts intermediate was synthesized through a nucleophilic substitution reaction between TsCl and PEG4k/5k. Its chemical structure was confirmed by ^1^H-NMR analysis, as shown in Figures S1 and S2. The conversion ratio was calculated by comparing the integrals of the aromatic proton peak at δ 7.35–7.82 ppm of TsCl with the methoxy proton peak at δ 3.40 ppm of PEG. The yields of PEG4k-Ts and PEG5k-Ts were 91% and 84%, respectively.

**Figure 2:**
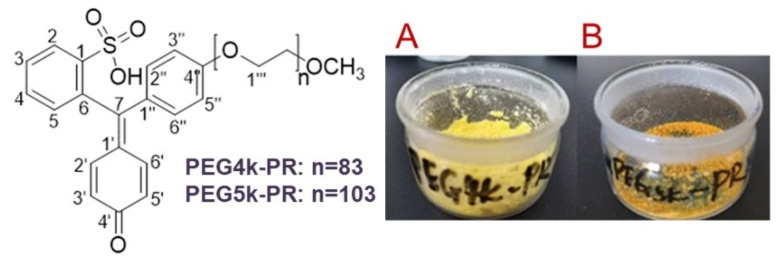
Structure and appearance of PEG4k-PR (**A**) and PEG5k-PR (**B**).

Afterwards, PEG4k/5k-PR was synthesized by substituting the PR to the Ts group of PEG-Ts. The ^1^H-NMR spectral data of PEG4k/5k-PR are shown in Figures S3 and S4, while the structural analysis is illustrated in Figure 2 and Table 2. Furthermore, the ^13^C-NMR spectra of PEG4k/5k-PR are shown in Figures S5 and S6. The yields of PEG4k-PR and PEG5k-PR were 47% and 39%, respectively. The purities of PEG4k-PR and PEG5k-PR were 95% and 96%, respectively.

**Figure 3:**
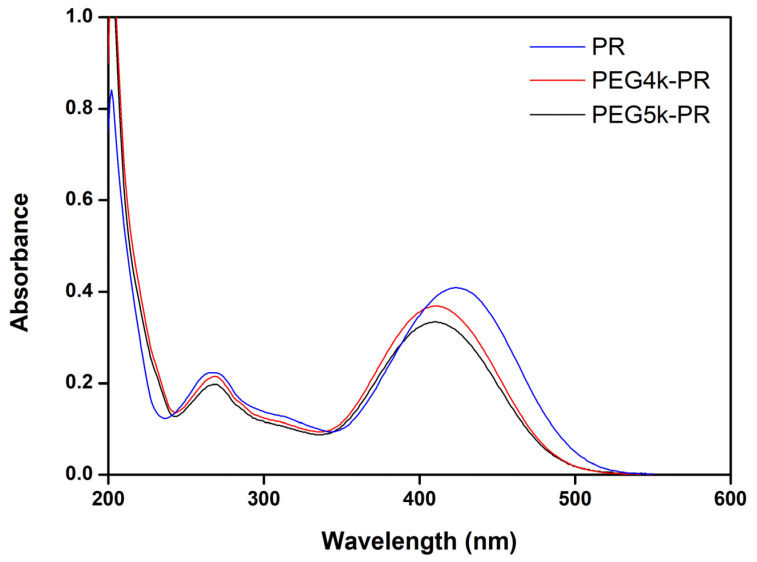
UV spectra of PR, PEG4k-PR, and PEG5k-PR.

**Figure 4:**
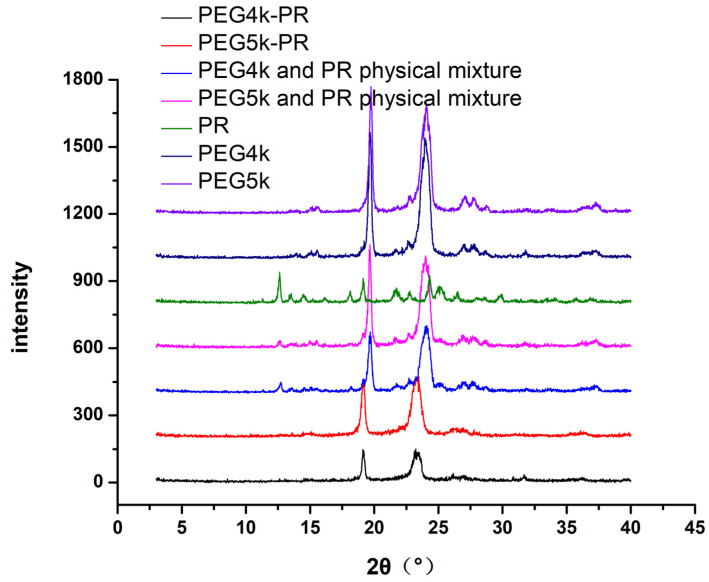
X-ray diffraction (XRD) spectra of PR, PEG4k/5k, PEG4k/5k-PR, and the physical mixtures of PEG4/5k and PR.

**Figure 5:**
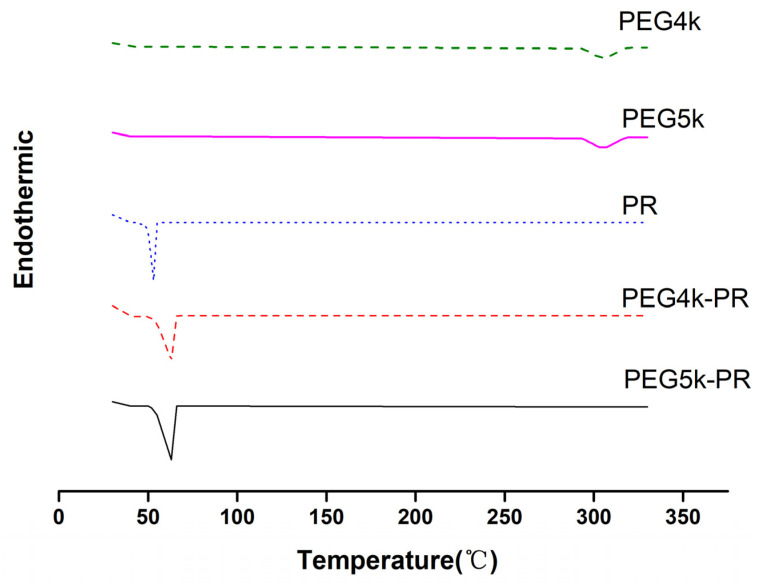
Differential scanning calorimetry (DSC) data of PR, PEG4k/5k, and PEG4k/5k-PR.

UV, XRD, and DSC assays were conducted to further assess the properties of PEG4k/5k-PR (Figure 3, Figure 4 and Figure 5). In the UV spectra, the novel markers PEG4k/5k-PR possessed absorption patterns similar to that of PR, with subtle blue shifts in the maximal absorption wavelength (410 nm). The results of XRD showed the obvious characteristic peaks of PEG4k/5k at 27°, and the multiple crystal diffraction peaks of PR disappeared in PEG4k/5k-PR, while they were simply overlapped in the physical mixture. In the DSC pattern of PEG4k/5k-PR, the endothermic peak of PEG4k/5k (at 304 °C) disappeared, and the endothermic peak of PR (51 °C) shifted to 63 °C. All of these results verified that a novel compound was successfully synthesized.

### 3.2. Stability of PR and PEG-PR

The concentration changes of PR, PEG4k/5k-PR, and the model drugs were calculated by a series of concentrations before and after the shaker bath. The results showed that all the percentages of concentration changes were within 2%, indicating that the stability of the marker was excellent and that their addition did not cause concentration fluctuations of the model drugs during perfusion.

### 3.3. Permeability of PR and PEG-PR in EGS

**Figure 6:**
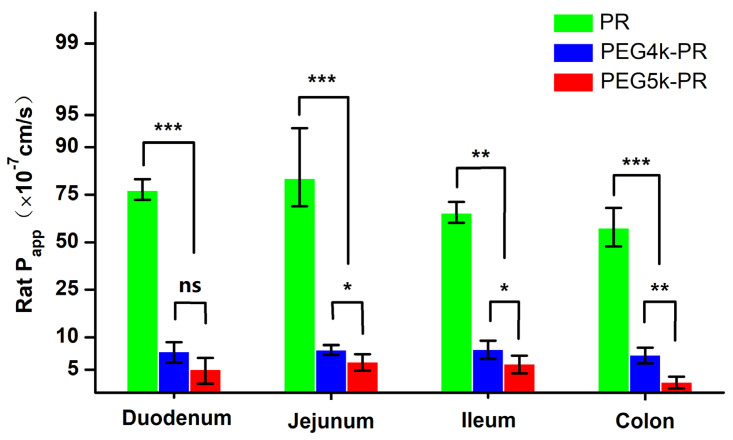
The apparent permeability (*P*_app_) of PR, PEG4k-PR, and PEG5k-PR at 56 μM in different intestinal segments (everted gut sac). Data are shown as mean ± SD, *n* = 4–6; * *p* < 0.05, ** *p* < 0.01 and *** *p* < 0.001.

The intestinal permeabilities of PR and PEG4k/5k-PR were investigated by everted gut sac measurements of different intestinal segments in rats; the results are shown in Figure 6. These results show that both PR and PEG4k/5k-PR could obviously be absorbed through intestinal membranes, with similar permeability trends: jejunum > duodenum > ileum > colon. However, the *P*_app_ values of PEG4k/5k-PR were 8–15 times lower than that of PR. Moreover, for the novel marker PEG-PR, the larger molecular weight resulting from the PEGylation in PEG5k-PR (with a larger molecular weight) induced a further significant reduction in *P*_app_.

**Figure 7:**
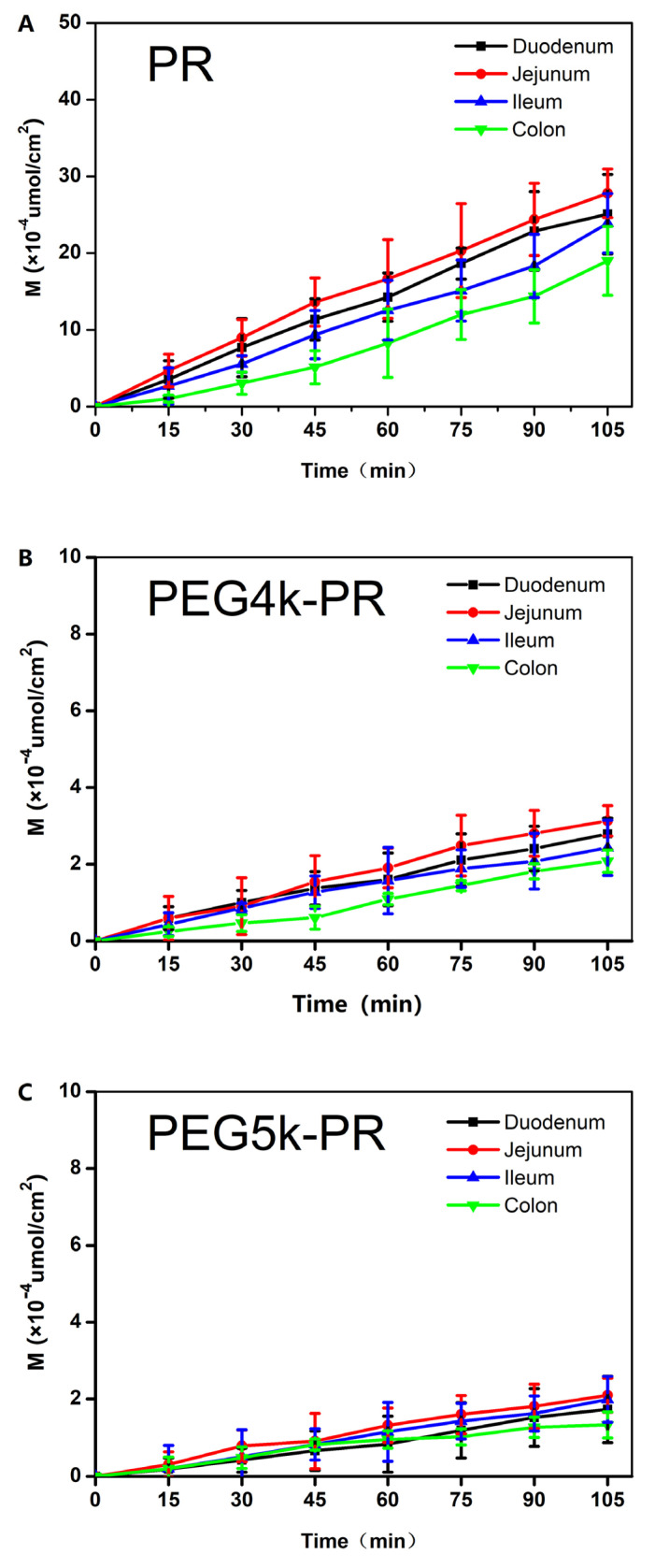
Cumulative intestinal permeable amount versus time of PR (**A**), PEG4k-PR (**B**), and PEG5k-PR (**C**) at 56 μM in different intestinal segments. Data are shown as mean ± SD, *n* = 3.

It has been reported that the urinary recovery of PR after 8 h in rat perfusion experiments can be up to about 4%, while the absorption of PEG4k is 0.5–4.2% within 60 min, as determined by detecting the amount of ^14^C-PEG4k in rat intestinal venous blood [25,26]. In this work, the profile of the cumulative intestinal permeable amount versus time (Figure 7) showed that the cumulative permeable percentage of PEG4k/5k-PR (0.2–0.7%) was profoundly lower than that of PR (3–5%) at a concentration of 56 μM in all intestinal segments. These results strongly demonstrated the nonabsorbable features of PEG4k/5k-PR, compared with PR.

**Figure 8:**
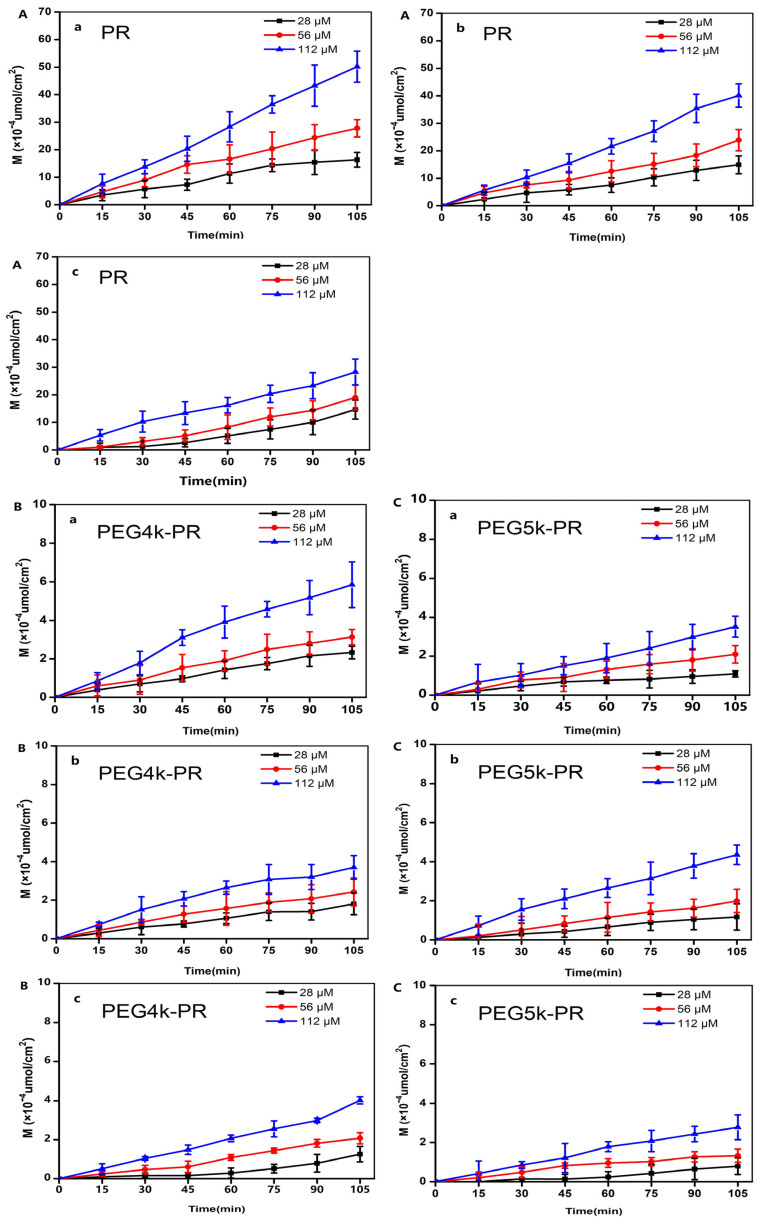
Cumulative intestinal permeable amount versus time of PR (**A**), PEG4k-PR (**B**), and PEG5k-PR (**C**) in the jejunum (**a**), ileum (**b**), and colon (**c**) at different concentrations (28–112 μM). Data are shown as mean ± SD, *n* = 3.

PR and PEG, which have been widely used for intestinal absorption studies in humans, are poorly absorbed by the intestines due to different mechanisms: PR has a low affinity to the gastric intestinal (GI) mucosa, while the size and shape of the high molecular weight PEG limit its intestinal transport [31]. In this work, different concentrations (28–112 μM) of different markers were employed to further explore the reasons and mechanisms of permeability, as illustrated in Figure 8 and Table 3. In different intestinal segments, the cumulative permeable percentage of PEG4k/5k-PR showed no significant difference with the changing concentration, indicating that the novel marker crossed the intestinal membrane by passive diffusion. However, the cumulative permeable percentage of 112-μM PR was twice as large as that with 28 μM in the colon, while the cumulative permeable percentage of PR did not show a significant concentration dependence in other intestinal segments. This phenomenon indicated that the membrane transport of PR involved other mechanisms. It has been reported that PR could be influenced through the addition of enhancers such as medium-chain glycerides and protease inhibitors; furthermore, Ca^2+^ and verapamil can also improve the absorption of PR [28,32,33]. These results illustrate that PEG-PR possesses significant advantages as a marker for studying the mechanism of drug intestinal transport.

In addition, it has been confirmed that the absorption of PEG is mainly due to the low molecular weight portions in one batch [25]. Here, we synthesized PEG-PR with different molecular weights to confirm and exclude the possibility of such an absorption. Higher PEGylation endowed PEG5K-PR with significantly lower permeability, although an absolutely nonabsorbed marker was not obtained. That cumulative permeable amount between PEG4k-PR and PEG5k-PR indicated that the permeable amount of the novel marker could be due to the low molecular weight portions of the markers. This provided a new strategy to decrease the permeability of the marker. However, the difficulty and cost of synthesis should not be neglected. Comparatively, PEG-PR with 4k and 5k molecular weight PEG was considered to be appropriate.

### 3.4. Evaluation of PR and PEG-PR with the In Situ Perfusion Model

**Figure 9:**
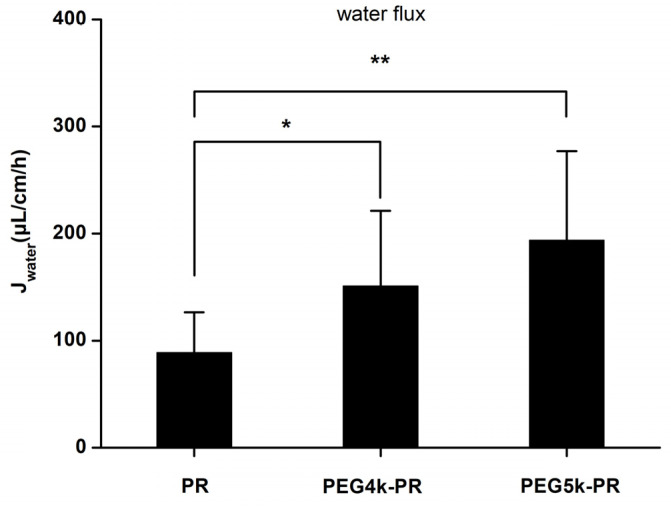
Water flux (*J*_water_) corrected by PR, PEG4k-PR, and PEG5k-PR at 56 μM during intestinal perfusion. Data are shown as mean ± SD, *n* = 6; * *p* < 0.05 and ** *p* < 0.01.

Water flux (*J*_water_) correction is essential in the calculation of *P*_eff_ when using an in situ perfusion model. However, self-absorption of the marker could lead to a decreased accuracy of the measured *J*_water_ value or even the appearance of a negative value. We next sought to determine the different abilities of PR and PEG-PR for water flux correction. The *J*_water_ (60–120 min) values corrected by PR, PEG4k-PR, and PEG5k-PR in the recirculation perfusion model were found to be 88.7 ± 37.9, 150.9 ± 70.3, and 193.8 ± 83.2 μL/cm/h, respectively (Figure 9). The *J*_water_ corrected by PEG4k-PR and PEG5k-PR were significantly larger (*p* < 0.05 and *p* < 0.01) than that corrected by PR, which reflected the more accurate correction due to the lower permeability of the novel marker.

The work conducted by Sutton et al. (2001) measured the average corrected water flux of different methods, including the PR (26.8 ± 49.2 μL/cm/h), PEG (34.9 ± 21.9 μL/cm/h), and gravimetric method (68.9 ± 28.2 μL/cm/h) in SPIP, which showed significant differences between the corrected water flux under different methods, where the absorption of PR and PEG may have been the reason for such differences [22]. Subsequent research conducted by Tugcu-Demiroz et al. (2014) validated PR (55 μL/cm/h) and the gravimetric method (80 μL/cm/h) for water flux in the Doluisio technique, where the results show that both methods had no difference [21,22]. Furthermore, Dahlgren et al. reported that the water flux corrected by gravimetric method for intra-abdominal and extra-abdominal were 232 ± 97 and 138 ± 70 μL/cm/h, respectively, in SPIP [34]. In spite of this variability, the evidence above suggests that absorption of the marker indeed occurs and even causes an underestimated water flux correction. It is worth noting that the calculation of *J*_water_ in previous methods was carried out by simply measuring the change between input and output of the water weight (Q) or marker concentration (C) during perfusion. Thus, the results mainly depended on the final sampling and ignored the real-time change during perfusion, which may have led to uncertain deviations [35]. Comparatively, the *J*_water_ values obtained by the linear regression analysis were more reasonable.

**Figure 10:**
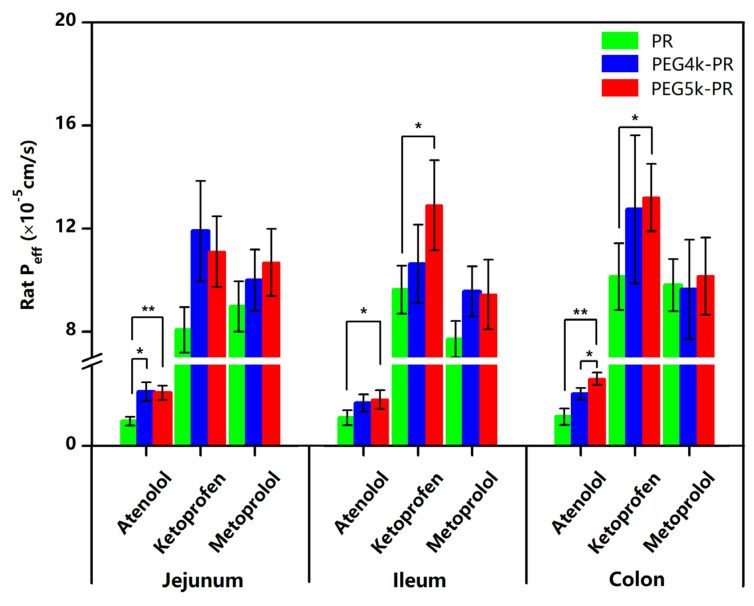
Effective intestinal membrane permeability (*P*_eff_) of atenolol, ketoprofen, and metoprolol corrected by PR, PEG4k-PR, and PEG5k-PR in different intestinal segments. Data are shown as mean ± SD, *n* = 3; * *p* < 0.05 and ** *p* < 0.01.

In order to validate the impact of the abovementioned water flux correction difference on the results of the in situ perfusion model, the *P*_eff_ values of atenolol, ketoprofen, and metoprolol were obtained using the recirculation intestinal segment perfusion technique (60–120 min) corrected by PR or PEG4k/5k-PR. The results (Figure 10) showed that the *P*_eff_ values of the three model drugs in different intestinal segments and corrected by different markers generally had the same trends, thus indicating the reliability of the data. However, the *P*_eff_ values of the three model drugs corrected by PEG5k-PR were larger than those corrected by PR in jejunum and ileum. Furthermore, for different model drugs, the *P*_eff_ values corrected by PEG4k/5k-PR and PR also showed differences, to various degrees. The *P*_eff_ values of atenolol (BCSⅢ) corrected by PEG4k/5k-PR had significant increases (*p* < 0.05 and *p* < 0.01) compared with that corrected by PR, while the *P*_eff_ values of metoprolol (BCSⅠ) had no difference, thus indicating that PEG-PR had a better ability for water correction, thus obtaining more accurate drug permeability parameter values; this capability plays an important role in the study of poorly absorbable drugs.

Drug permeability depends on the intestinal site as the drug is absorbed through different intestinal segments with different pH values, effective surface areas, enzymatic activities, membrane transport expression, and blood circulation. Drug permeability in the colon has recently attracted increased attention, as the majority of controlled release drugs designed for longer time dosage release are absorbed in the colon [36]. The in situ perfusion model supplies permeability estimates with convenient and reliable means, and, so, many drug permeability studies using SPIP or the closed loop method have been published and compared [17]. However, to the best of our knowledge, no adequate relevant report about the recirculation method has been published. Therefore, the *P*_eff_ values of atenolol, ketoprofen, and metoprolol were investigated in different intestinal segments with recirculation perfusion corrected by the novel marker proposed in this work. In addition, the *P*_eff_ values of the three model drugs in the complete small intestines of rats were obtained using different markers, as presented in Table 4.

Atenolol, which is absorbed by a paracellular route, is a typical drug belonging to BCS Ⅲ. Its *P*_eff_ values corrected by PEG5k -PR in jejunum, ileum, and the colon were 2.6 × 10^−5^, 2.3 × 10^−5^, and 3.3 × 10^−5^ cm/s, respectively. The *P*_eff_ value of metoprolol was set as the minimum boundary for a highly permeable drug, and the corresponding values were 10.7 × 10^−5^, 9.4 × 10^−5^, and 10.2 × 10^−5^ cm/s, respectively. In addition to the comparison with metoprolol, the drugs with *P*_eff_ values lower than 4.2 × 10^−5^ cm/s were classified as poorly permeable drugs according to the research reported by Zakeri-Milani et al. [37]. For some poorly absorbed drugs, the *P*_eff_ values should be carefully supplied, as its calculation is more likely to be influenced by the (non-negligible) absorption of the marker. Furthermore, the inaccurate permeability estimation of drugs having a narrow therapeutic window (e.g., digoxin) by the in situ perfusion model could lead to the clinically relevant concentration obtained presenting either an inadequate response or toxicity [38].

Ruiz-Picazo et al. (2017) evaluated the *P*_eff_ of atenolol using the Doluisio method in the jejunum, ileum, and colon, which were 2.27 × 10^−5^, 1.63 × 10^−5^, and 2.11 × 10^−5^ cm/s, respectively; the corresponding *P*_eff_ values of metoprolol and ketoprofen were 6.85 × 10^−5^, 9.05 × 10^−5^, and 8.14 × 10^−5^ cm/s and 6.9 × 10^−5^, 3.65 × 10^−5^, and 22.8 × 10^−5^ cm/s, respectively [39]. The intestinal segment *P*_eff_ values of ketoprofen demonstrated an unexplainable difference in the colon. In this work, the *P*_eff_ values of ketoprofen (11.1 × 10^−5^ and 12.9 × 10^−5^ cm/s, respectively) in the ileum and colon corrected by PEG5k-PR and PR displayed significant differences, while those in the duodenum corrected by different markers showed no difference. In the study of the everted gut sac, the permeability of the PR showed a concentration dependence in the colon. All of these results indicate that there is an unknown interplay between ketoprofen and PR during intestinal transport.

The in situ perfusion model in rats represents an optimal means to study the permeability and absorptive kinetics of drugs, whereby the obtained assessment is based on the disappearance of the drug in the lumen. The blood and nerves are maintained in the experimental animal to mimic in vivo conditions, which leads to the accurate prediction of human drug permeability [40,41]. The recirculation method is a reliable model for estimating poorly absorbed drugs, as it can supply a longer retention time to magnify the absorption. However, water absorption becomes correspondingly pronounced, which affects the accuracy of determination. Compared with PR, the novel PEG-PR marker has a better capability for water flux correction in this model.

## 4. Conclusions

The novel marker PEG4k/5k-PR was shown to be stable and less absorbable in comparison with PR. Its nonabsorbable effect may be related to the length of the PEG chain (molecular weight), and the intestinal absorption was not found to be significantly dependent on the concentration. The evaluation of its application in estimating the model drug permeability by using the recirculation perfusion method demonstrated that PEG-PR can provide a more accurate assessment of the drug permeability, especially for poorly absorbable drugs. Therefore, PEG-PR is a promising marker with the potential application for intestinal permeability studies using the in situ perfusion model in rats.

## Figures and Tables

**Table 1:** HPLC conditions of the compounds (with isocratic elution).

Compound (Internal Standard)	Mobile Phase (Aqueous: Organic)	λ (nm)	Retention Time (min)
Phenol red (cresol red)	A: Acetonitrile (40:60, *v*/*v*)	430	8.1 (13.2)
PEG4k/5k-PR (cresol red)	A: Acetonitrile (46:54, *v*/*v*)	410	5.2 (7.9)
Atenolol (ranitidine)	B: Acetonitrile (90:10, *v*/*v*)	226	7.8 (9.3)
Ketoprofen (ibuprofen)	B: Acetonitrile (45:55, *v*/*v*)	220	7.5 (17.3)
Metoprolol (phenytoin sodium)	C: Methanol (56:44, *v*/*v*)	226	7.6 (9.4)

A: pH 3.0 phosphate buffer containing 5-mM tetrabutylammonium bromide, B: pH 3.0 phosphate buffer, and C: pH 3.0 phosphate buffer containing 0.4-mL triethylamine (TEA). PEG: polyethylene glycol.

**Table 2:** ^1^H-NMR analyses of PEG4k/5k-PR.

NO.	PEG4k-PR	PEG5k-PR
	δH	δH
2	7.90 (1H, dd J = 7.6, 1.1 Hz)	7.90 (1H, dd J = 7.8, 1.1 Hz)
3	7.42 (1H, t, J = 7.3 Hz)	7.42 (1H, t, J = 6.9 Hz)
4	7.37 (1H, t, J = 7.3 Hz)	7.37 (1H, t, J = 7.4 Hz)
5	6.97 (1H, overlapped)	6.98 (1H, overlapped)
2′	7.48 (1H, dd, J = 9.9, 2.5 Hz)	7.48 (1H, dd, J = 10.0, 2.6 Hz)
3′	6.32 (1H, dd, J = 9.9, 1.8 Hz)	6.32 (1H, dd, J = 10, 2.1 Hz)
5′	6.10 (1H, dd, J = 9.9, 1.8 Hz)	6.10 (1H, dd, J = 10.0, 2.1 Hz)
6′	6.85 (1H, dd, J = 9.9, 2.5 Hz)	6.85 (1H, dd, J = 10.0, 2.6 Hz)
2′’	7.35 (1H, d, J = 8.7 Hz)	7.33 (1H, d, J = 8.8 Hz)
3′’	6.97 (1H, d, J = 8.7 Hz)	6.97 (1H, d, J = 8.8 Hz)
5′’	6.97 (1H, d, J = 8.7 Hz)	6.97 (1H, d, J = 8.8 Hz)
6′’	7.35 (1H, d, J = 8.7 Hz)	7.32 (1H, d, J = 8.7 Hz)
1′’’	4.14 (2H, t, J = 4.5 Hz)	4.14 (2H, t, J = 4.8 Hz)
-(OCH_2_CH_2_O)_n_	3.38–3.74 (457H, overlapped)	3.38–3.74 (504H, overlapped)
-OCH_3_	3.25 (3H, s)	3.25 (3H, s)

**Table 3:** Apparent permeability coefficients (*P*_app_) (cm/s) and cumulative permeable percentage of PR, PEG4k-PR, and PEG5k-PR.

	Conc.	Jejunum		Ileum		Colon	
	(μM)	*P*_app_ (×10^−6^ cm/s)	W (%)	*P*_app_ (×10^−6^ cm/s)	W (%)	*P*_app_ (×10^−6^ cm/s)	W (%)
**P**	28	7.68 ± 2.04	4.44 ± 1.98	6.17 ± 2.17	3.55 ± 1.50	4.07 ± 1.33	2.50 ± 0.82
	56	8.36 ± 3.11	4.92 ± 1.12	6.77 ± 2.32	4.23 ± 1.38	5.83 ± 1.9	3.36 ± 0.79
	112	10.42 ± 2.58	5.77 ± 1.70	8.90 ± 1.65	5.27 ± 2.31	8.57 ± 3.54	5.18 ± 1.22
**PEG4k-PR**	28	0.87 ± 0.15	0.81 ± 0.11	0.61 ± 0.13	0.32 ± 0.05	ND *	ND *
	56	0.83 ± 0.21	0.55 ± 0.07	0.66 ± 0.37	0.42 ± 0.13	0.51 ± 0.08	0.36 ± 0.05
	112	1.36 ± 0.32	0.54 ± 0.1	0.97 ± 0.28	0.63 ± 0.19	0.65 ± 0.14	0.44 ± 0.13
**PEG5k-PR**	28	0.55 ± 0.16	0.39 ± 0.15	0.69 ± 0.13	0.42 ± 0.16	ND *	ND *
	56	0.60 ± 0.08	0.37 ± 0.07	0.57 ± 0.28	0.36 ± 0.10	0.39 ± 0.07	0.24 ± 0.04
	112	0.54 ± 0.23	0.35 ± 0.08	0.61 ± 0.16	0.39 ± 0.04	0.41 ± 0.18	0.25 ± 0.07

* Not detected.

**Table 4:** Effective intestinal membrane permeability (*P*_eff_) (cm/s) values of atenolol, ketoprofen, and metoprolol corrected by different markers in rat complete small intestines using the recirculation perfusion method. Data are shown as mean ± SD, *n* = 3.

Drug	*P*_eff_ (×10^−5^ cm/s)		
	PR	PEG4k-PR	PEG5k-PR
Atenolol	0.49 ± 0.10	1.06 ± 0.24	1.38 ± 0.34
Ketoprofen	6.76 ± 2.43	7.47 ± 1.04	8.63 ± 1.26
Metoprolol	5.11 ± 0.85	5.06 ± 0.57	5.29 ± 1.49

## Supplementary Materials

The following are available online. Figures S1–S6: ^1^H and ^13^C NMR spectrum of PEG4k/5k-TsCl and PEG4k/5k-PR. Table S1: Concentration changes of all compounds before and after the shaker bath experiment. Data are shown as mean ± SD, *n* = 3. Figures S7–S12: Calibration lines of PR, PEG4k/5k-PR atenolol, ketoprofen and metoprolol in Krebs–Ringer buffer.
