# Supplementary material for: Synthesis and Evaluation of PEG-PR for Water Flux Correction in an In Situ Rat Perfusion Model
Source: Molecules. 2020 Nov 4;25(21):5123. doi: 10.3390/molecules25215123 (PMC7662639; doi:10.3390/molecules25215123)
Supplement: Supplementary file 1 [file molecules-25-05123-s001.pdf]

Supplementary Materials:

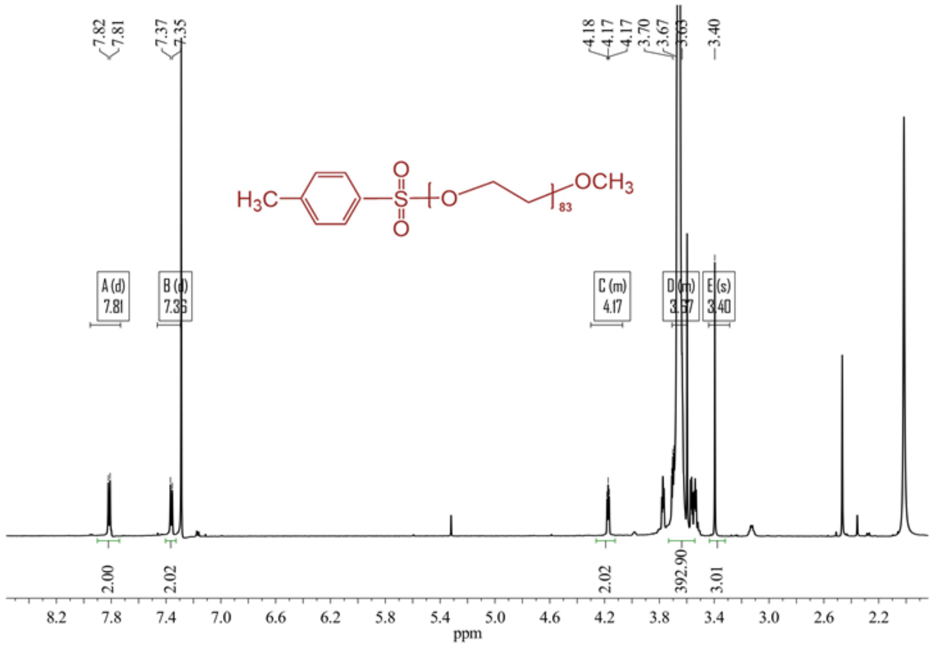

Figure S1. ^{1}H-NMR spectrum of PEG4k-TsCl.

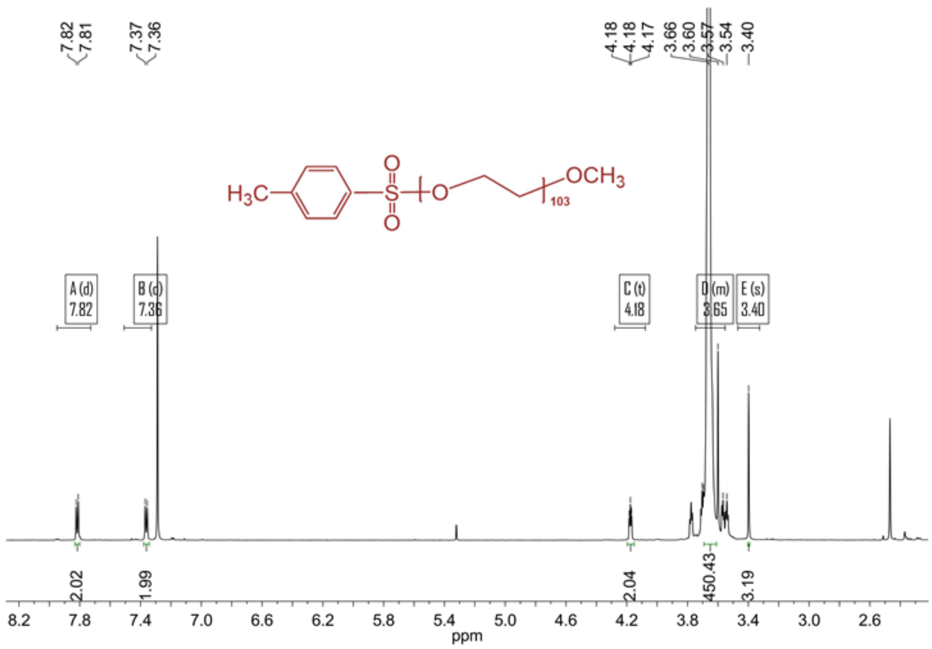

Figure S2. ^{1}H-NMR spectrum of PEG5k-TsCl.

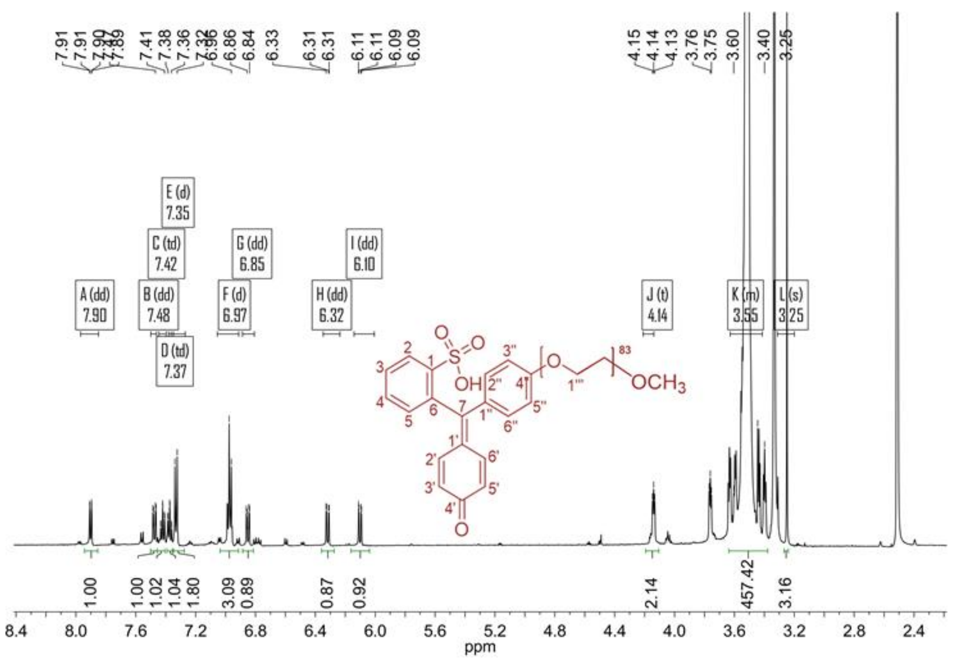

Figure S3. ^{1}H-NMR spectrum of PEG4k-PR.

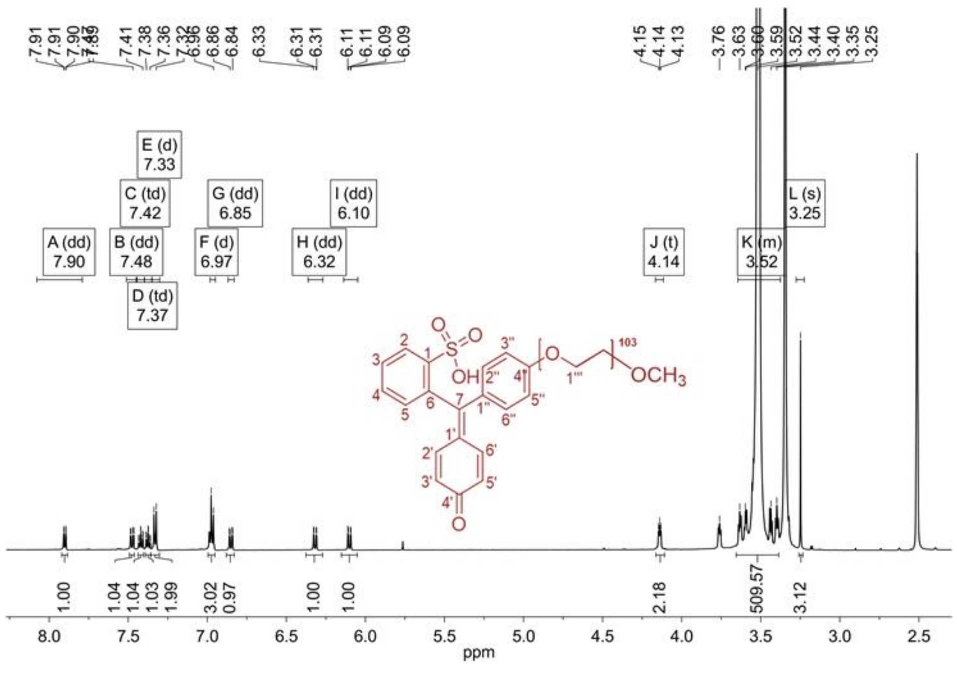

Figure S4. ^{1}H-NMR spectrum of PEG5k-PR.

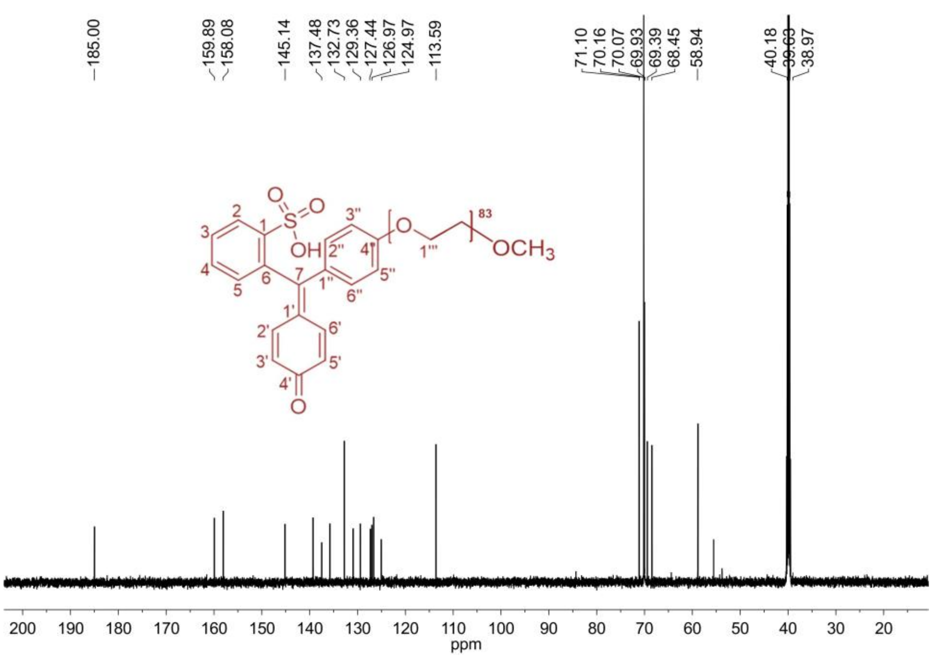

Figure S5. ^{13}C-NMR spectrum of PEG4k-PR.

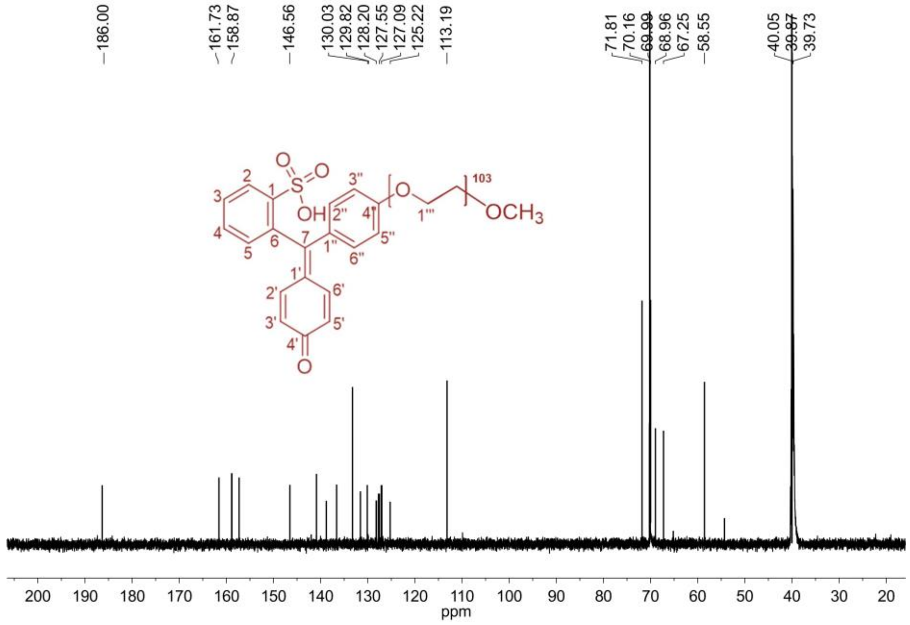

Figure S6. The ^{13}C-NMR spectrum of PEG5k-PR.

Table S1. Concentration changes of all compounds before and after the shaker bath experiment. Data are shown as mean ± SD, n = 3.

**Compound**	**Initial**		**Ending (120 min)**		**Percentage of concentration change**
	As/Ai	Conc. (μM)	As/Ai	Conc. (μM)	
PR	0.32 ± 0.01	56.90 ± 1.10	0.31 ± 0.01	56.11 ± 1.57	1.38%
PEG4kPR	0.54 ± 0.01	54.87 ± 2.44	0.54 ± 0.01	54.29 ± 0.61	1.07%
PEG5k-PR	0.87 ± 0.01	56.47 ± 0.65	0.86 ± 0.01	55.58 ± 0.37	1.57%
Atenolol	0.71 ± 0.01	105.19 ± 0.96	0.71 ± 0.01	105.93 ± 0.61	0.75
Ketoprofen	1.11 ± 0.02	101.27 ± 1.22	1.09 ± 0.01	100.28 ± 0.96	0.97
Metoprolol	0.79 ± 0.02	103.62 ± 2.29	0.76 ± 0.01	102.12 ± 0.58	1.46

As: HPLC peak area of compound.

Ai: HPLC peak area of internal standard.

Percentage of concentration change: (Initial Conc.-Ending Conc.) / Initial Conc.

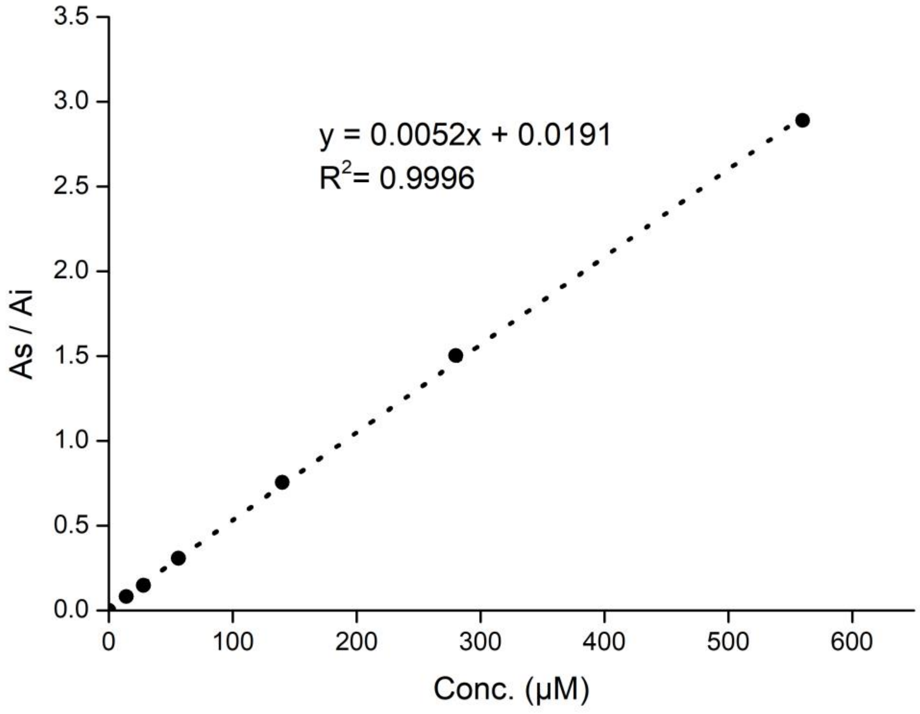

Figure S7. Calibration line of PR (MW = 354) in Krebs–Ringer buffer.

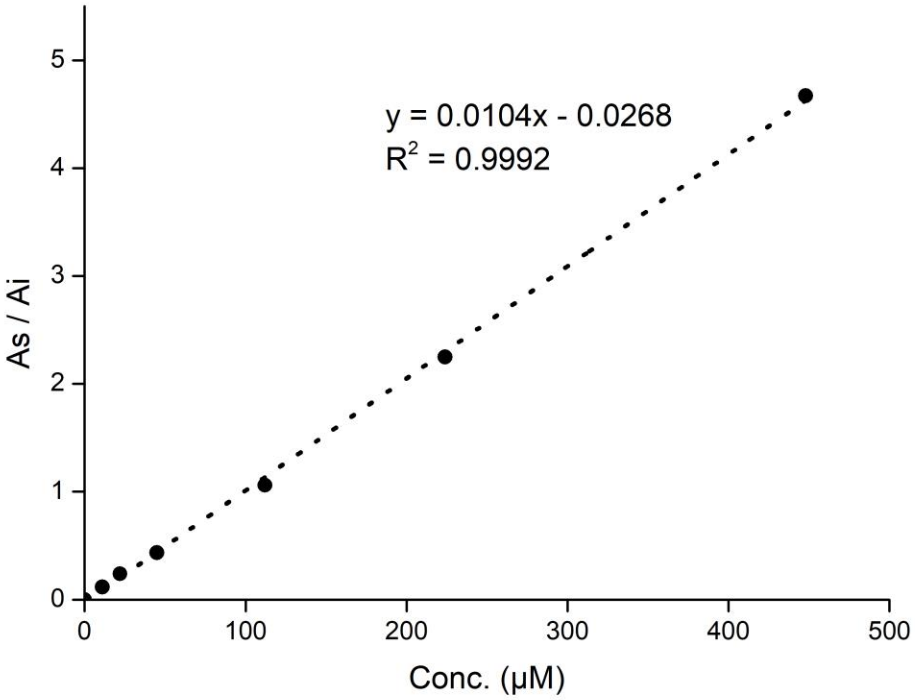

Figure S8. Calibration line of PEG4k-PR (MW = 4354) in Krebs–Ringer buffer.

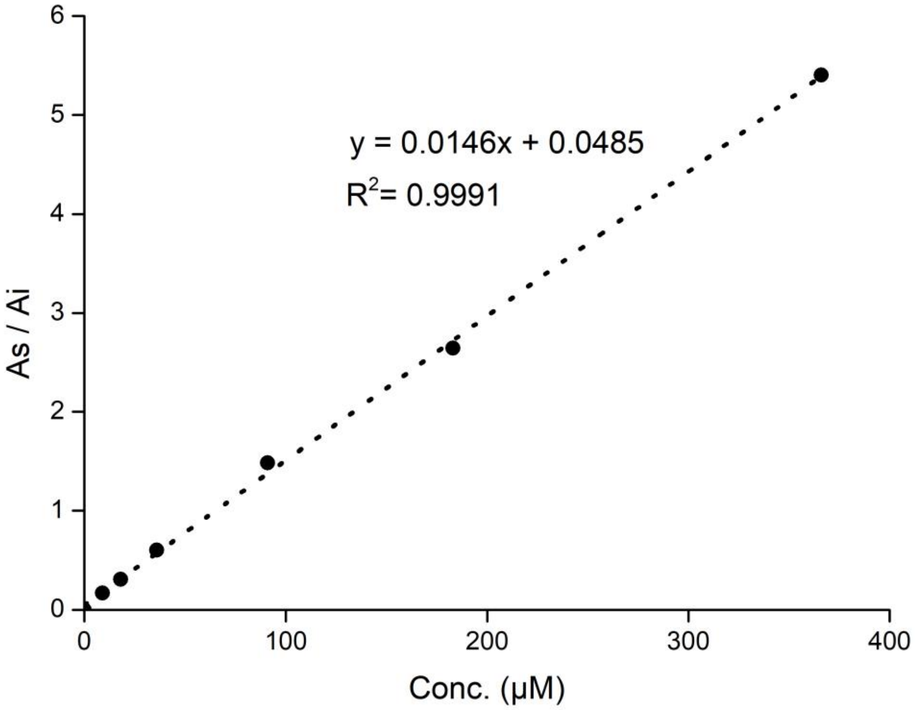

Figure S9. Calibration line of PEG5k-PR (MW = 5354) in Krebs–Ringer buffer.

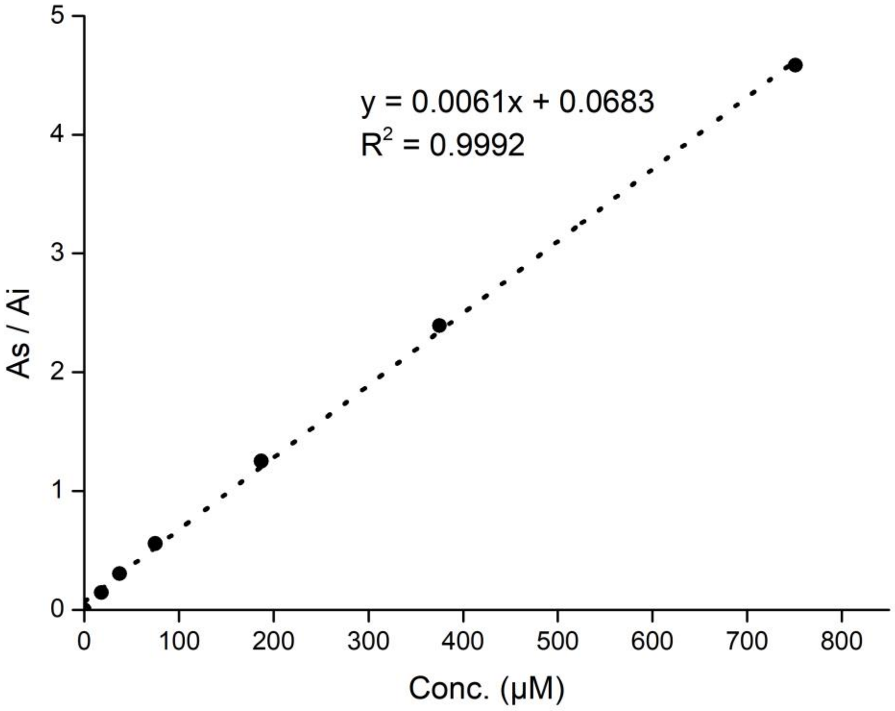

Figure S10. Calibration line of atenolol (MW = 266) in Krebs–Ringer buffer.

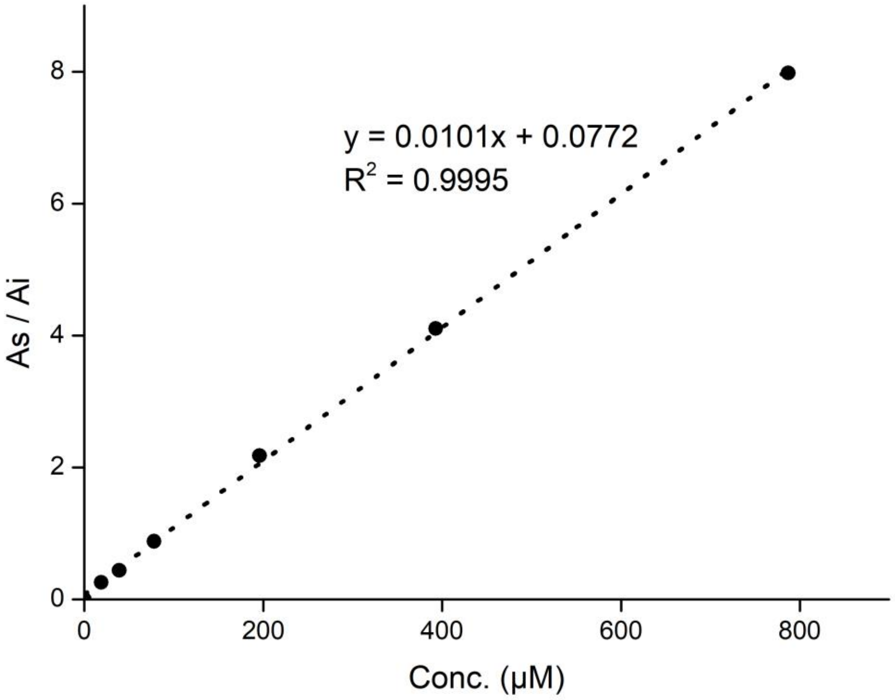

Figure S11. Calibration line of ketoprofen (MW = 254) in Krebs–Ringer buffer.

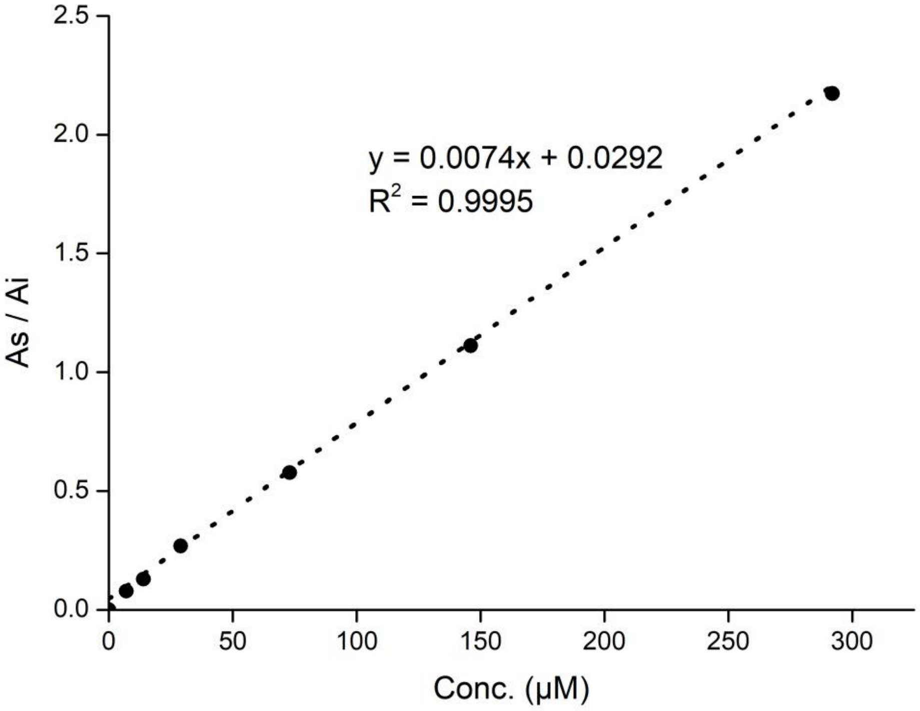

Figure S12. Calibration line of metoprolol (MW = 684) in Krebs–Ringer buffer.
